# Editorial: Design, modeling and control of kinematically redundant robots

**DOI:** 10.3389/frobt.2024.1399217

**Published:** 2024-04-08

**Authors:** Yangming Lee, Ivan Virgala, S. M. Hadi Sadati, Egidio Falotico

**Affiliations:** ^1^ RoCAL Lab, Rochester Institute of Technology, Rochester, NY, United States; ^2^ Applied Robotics and Mechatronics Laboratory, Technical University of Košice, Košice, Slovakia; ^3^ Robotics and Vision in Medicine (RViM) Lab, King’s College London, London, United Kingdom; ^4^ BioRobotics Institute, Sant’Anna School of Advanced Studies, Pisa, Italy

**Keywords:** redundant robot, modeling, design, control, robotics

Redundant robots, which were originally inspired by biological redundant structures, have more joints and movable axes than the minimum required to reach a specific position and orientation in their workspace (Spong et al., 2020). Redundancy improves flexibility and dexterity, thereby improving obstacle avoidance and fault tolerance, and leads to improved environmental adaptability and system robustness. However, redundant mechanical structures not only increase the difficulty of mechanical design, but also increase the number of actuators and sensors, and pose challenges in control and path planning. Recent research has deeply integrated machine learning methods with the design, modeling, and control of redundant robots to reduce modeling difficulty, improve control efficiency, and enhance system reliability (Jin et al., 2018; Wang et al., 2021; Li, 2023). This Research Topic assembles some representative works, including the discussion of existing challenges in redundant soft robot modeling (Sadati et al.), and latest breakthrough in using continuum robots to improve dexterity (Grassmann et al.; Yang et al.), and solving the control and planning problems with learning techniques (Yang et al.; Ma et al.).

Sadati et al. reviewed and compared the techniques of Reduced-Order Modeling (ROM) and Model Order Reduction (MOR) that improve the control efficiency of Soft Robots. Controlling soft robots is difficult because their shapes are complex and their configuration spaces are high-dimensional (Whitesides, 2018). Geometrically exact modeling approaches, like Cosserat rod and Finite Element Methods (FEM), are computationally expensive for real-time control (Till et al., 2019). ROM and MOR can address these challenges by creating lower-dimensional models of the soft robot. ROM uses simplifying assumptions to create a lower-dimensional model, while MOR reduces the state-space dimension of a high-fidelity FEM-based model. The work provides an in-depth survey of ROM and MOR techniques for the continuum and soft robotics and can provide aid in selecting proper models for specific tasks.

Grassmann et al. presented a fully actuated segment (FAS) design for tendon-driven continuum robots to verify the hypothesis that utilizing all degrees of freedom significantly improves motion capabilities, follow-the-leader (FTL) deployment, and position and orientation capabilities. Being different from rigid surgical robots or other tendon-driven flexible robots, the actuated twist of the proposed system is implemented by reconsidering spacer disks and leveraging the design of a concentric tube continuum robot. It translated the robot backbone and used floating spacer disks to implement variable segment lengths (Li et al., 2019; Anderson et al., 2024; Rosen et al., 2017). By simply rotating the backbone, it achieves variable tendon routing, therefore, it exploits four DOF for one segment; bending in two planes, translation, and rotation.

Yang et al. also studied continuum robots. This work utilized the flexibility and adaptability of the continuum manipulator to navigate complex spaces and conform to the shape of the cracks for precise repair. Furthermore, by integrating the continuum arm with an unmanned aerial vehicle (UAV), the system can detect and repair structural damage for building safety and cost-effectiveness. The detection and repair of structural damages were automatized by artificial intelligence. A convolutional neural network is trained to represent the structural feature of the crack with a centerline. Furthermore, a multi-layer perceptron neural network (MLPNN) estimates the length of the bending tubes for guiding the continuous deposit of the putty material to fill the microscopic crack.

Ma et al. studied the dynamics and control of maintaining a squirrel’s head facing the landing spot, while its other body segments tumble in the air. The work developed a simplified 2D multibody dynamics model with body segment collision constraints of a squirrel and further applied two very different control methods to reproduce the observed squirrel behavior. The first control method is classical: it plans a reference motion trajectory, represents the squirrel motion behavior, and applies a PD feedback controller to track the planned reference trajectory. The second control method utilized reinforcement learning (RL) techniques (Kaelbling et al., 1996): it trains a deep neuron-network-based control policy based on the observations. Comparatively, the RL method performs better in terms of closer to expected behavior and robustness against sensor errors, however, shows more variant joint motions with respect to noisy input data near the landing time. Meanwhile, the RL method do not need to plan a reference trajectory, and thus the method would suit more to the natural environment and lead to more natural outcomes.

The works covered in the Research Topic provide valuable insights and resources for researchers and engineers working on redundant robots, help them to deeply understand the challenges of redundant robot control, select proper modeling methods, and improve applications in surgery and construction.
